# David Abrahamson, MBE, FRCPI, FRCPsych

**DOI:** 10.1192/bjb.2024.99

**Published:** 2025-06

**Authors:** Alan Eppel

**Figure:**
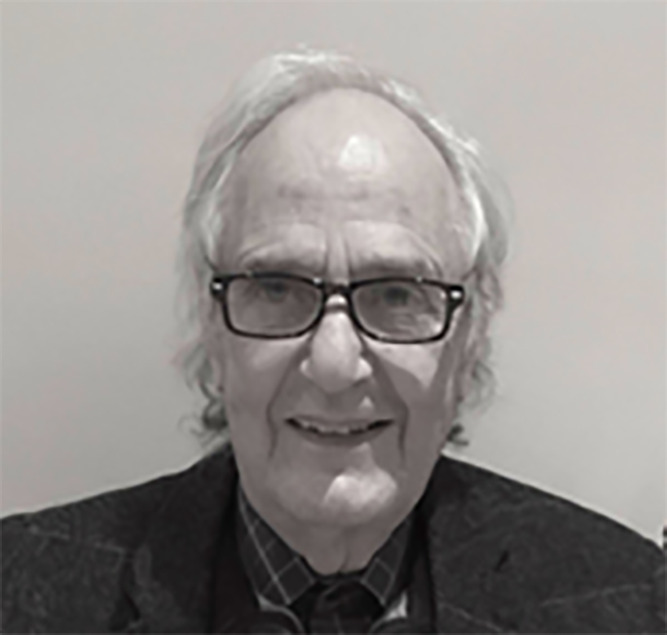

Formerly Consultant Psychiatrist, Goodmayes Hospital, Ilford

While training at the Maudsley Hospital in the 1960s, David Abrahamson, who died on 22 August 2024, aged 92, came to believe that there was too much distance between psychiatrists and their patients. He saw this as often diminishing the humane nature of the treatment relationship. He also recognised a large divide between teaching hospitals, such as the Maudsley, and mental hospitals. He felt that long-term patients were treated as ‘second class’. This theme was to become a motivating force in his life's work.

In 1971, he was appointed as a consultant at Goodmayes Hospital in East London. There he had responsibility for two acute in-patient units, one unit for ‘disturbed’ patients and 300 long-stay patients. He also worked in out-patient clinics and taught junior doctors. It was a heavy workload that he took on willingly. He was highly committed to the National Health Service (NHS) and refused to engage in any form of private practice. While in post, he undertook a Department of Health funded research project into 491 long-stay patients who had been in hospital for 20 to more than 50 years. He demonstrated that many of these individuals did not exhibit significant deterioration from the time of their admission and had long stable periods. This indicated to him their potential for rehabilitation and resettlement. The findings were presented in a final report to the Department of Health and Social Security and incorporated into an influential peer-reviewed article.^1^

At Goodmayes David led a multidisciplinary rehabilitation team composed of community psychiatric nurses, psychologists, occupational therapists and a speech and language therapist. The team practised an empathic, patient-centred approach. Deinstitutionalisation of the mentally ill in the USA, the UK and Canada had been largely unsuccessful, resulting in homelessness and despair. In contrast, David and his team were successful in moving long-stay patients into the community. Of prime importance in his approach was working with a multidisciplinary rehabilitation team and close liaison with local housing authorities. When long-stay patients moved into supported housing they missed their friends from the wards. David and his team set up a weekly social club, which he attended. The social club increased the opportunities for new relationships and acquaintances in the community. He recognised the existence of meaningful social networks on long-stay units and the importance of maintaining these when moving into community housing. Over the ensuing three decades multiple housing units with varying formats were established in collaboration with housing authorities, particularly Springboard, of which he became a trustee. It was for his work with long-stay patients and their move into community housing that he was awarded an MBE in 2002.

David Abrahamson was born in Dublin on 29 October 1932 under rather remarkable circumstances. He was the second of a pair of very premature twins delivered with great difficulty at the Portobello Nursing Home. Some days after the delivery there was a fire in the building. David, his twin brother Max and their mother had to be rapidly evacuated. At home the twins were kept in shoeboxes inside a chest of drawers – incubators were not available! David always recounted these events with a sense of resilience. He was the fifth child of Leonard and Tillie Abrahamson. In addition to his twin brother, he had two older brothers and one older sister. His father was Professor of Medicine at The Royal College of Surgeons of Ireland. After school, David trained and practised as a veterinarian and then qualified as a doctor at Trinity College, Dublin.

After medical training, David's interest in psychiatry was piqued when, as a senior house officer at the North Middlesex Hospital, he was impressed with the sense of camaraderie among the psychiatric patients. This was one of the factors that led him to appreciate the importance of social networks.

Possessed of great humanity, intellect and compassion, he was dedicated to the care of the most seriously psychiatrically ill patients. He was beloved by his family and devoted to his wife Valerie (née Duke Cohan), whom he married in 1961. Valerie had been a ballerina with the Royal Ballet and the Festival Ballet Companies and taught dance for several years. They had two daughters, Leonie and Vanessa. David took great interest in their careers and both of them recall him helping them with proofreading their manuscripts. He had a subtle dry wit which endeared him to his extended family and friends.
