# David Abrahamson, MBE, FRCPI, FRCPsych - ERRATUM

**DOI:** 10.1192/bjb.2024.109

**Published:** 2025-06

**Authors:** Alan Eppel

This obituary originally published with an incorrect former affiliation for David Abrahamson. All versions of the article have been updated.

## References

[ref1] Eppel A. David Abrahamson, MBE, FRCPI, FRCPsych. *BJPsych Bulletin*. Published online 2024:1-1.10.1192/bjb.2024.10939659230

